# Immunocompetent but Not Immune: A Prolonged Course of Cryptococcal Meningitis

**DOI:** 10.7759/cureus.103761

**Published:** 2026-02-17

**Authors:** Carlos Fagundo, Abdul Rahman Omari, Mauricio Valenzuela

**Affiliations:** 1 Internal Medicine, BayCare Medical Group, Tampa, USA

**Keywords:** cryptococcal meningitis, cryptococcous neoformans, fungal infection, immunocompetent, meningitis

## Abstract

*Cryptococcus neoformans* is an encapsulated yeast that typically causes cryptococcal meningitis (CM) in immunocompromised hosts. However, CM can rarely occur in immunocompetent individuals, often with atypical or insidious presentations. Persistently positive cerebrospinal fluid (CSF) cultures despite guideline-directed antifungal therapy are uncommon and pose significant management challenges. In this case report, we describe a previously healthy 40-year-old immunocompetent woman who presented with seizures, progressive headaches, and elevated intracranial pressure (ICP). CSF studies confirmed CM with positive cryptococcal antigen and cultures. She was treated with liposomal amphotericin B and flucytosine and later transitioned to high-dose fluconazole due to medication intolerance. Despite >6 weeks of antifungal therapy, serial CSF cultures remained persistently positive through hospital day 56, with clearance achieved only on day 72. ICP management required serial lumbar punctures, temporary lumbar drainage, and ultimately ventriculoperitoneal (VP) shunt placement on day 80. The patient was discharged on prolonged fluconazole consolidation therapy with resolution of headaches but residual right-eye visual deficits. This case highlights the potential for CM in immunocompetent hosts with protracted culture positivity despite guideline-directed antifungal therapy. Early recognition of antifungal intolerance, aggressive ICP management, and timely surgical intervention may be critical for optimizing outcomes in such complex cases.

## Introduction

*Cryptococcus neoformans* is an encapsulated, opportunistic yeast that causes cryptococcosis, a potentially life‑threatening invasive fungal disease. The organism is found in soil contaminated with avian droppings and decaying wood and is acquired primarily through inhalation of desiccated yeast cells [1]. Following inhalation, *C. neoformans* can establish latent infection or disseminate hematogenously. It commonly invades the central nervous system (CNS), where it causes meningoencephalitis [1,2].

Cryptococcal meningitis (CM) most commonly occurs in patients with advanced HIV infection, solid‑organ transplantation, or other causes of severe cellular immunodeficiency [1,3]. Disease in immunocompetent hosts does occur and may present insidiously, leading to diagnostic delay and increased risk of neurologic morbidity [4,5]. Clinical manifestations include subacute headache, fever, altered mental status, and visual changes. Elevated intracranial pressures (ICPs) are associated with increased morbidity and mortality [1,6].

Current guidelines recommend induction therapy with amphotericin B plus flucytosine for at least four to six weeks, followed by consolidation and maintenance therapy with fluconazole, alongside aggressive ICP control when needed [1,2,6]. We present the case of an immunocompetent woman with CM and positive cerebrospinal fluid (CSF) cultures for over 70 days despite guideline‑directed antifungal therapy. The patient required a prolonged course of induction medication and ventriculoperitoneal (VP) shunting for definitive ICP control. 

## Case presentation

We present a previously healthy 40-year-old woman with obesity (BMI 36.7 kg/m²) and a history of bipolar disorder, anxiety, depression, post-traumatic stress disorder (PTSD), schizophrenia, hypertension, and tachycardia who presented to the emergency department with a three-week history of worsening headaches, generalized weakness, dizziness, and recurrent falls. The patient has no history of chronic corticosteroid use, immunosuppressive therapy, diabetes mellitus, or chronic liver disease. On the day of admission, she sustained a fall with minor head trauma but no loss of consciousness. In the emergency department, she developed a generalized tonic-clonic seizure that terminated after two doses of intravenous lorazepam (2 mg each). 

**Figure 1:**
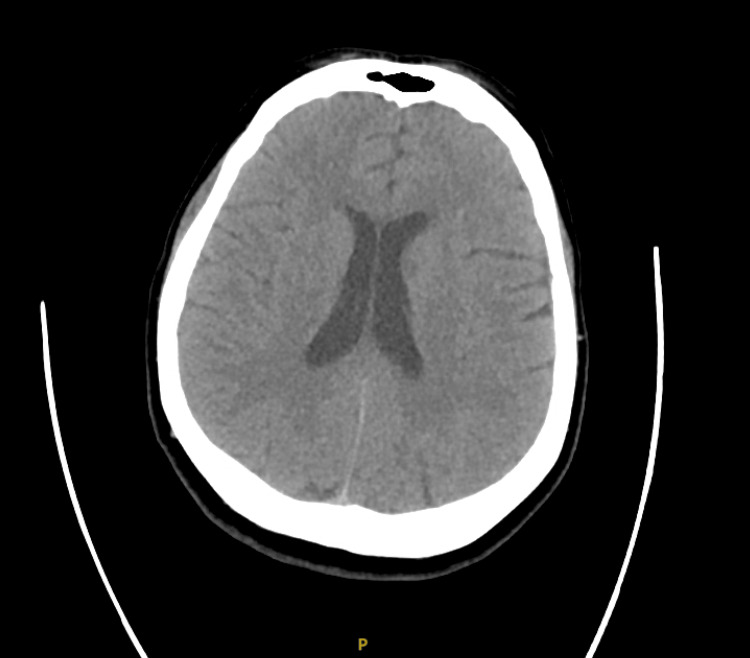
Computed tomogrpahy (CT) without contrast of the head, transverse view. Normal brain formation. There is no hydrocephalus, acute intracranial mass, mass effect or midline shift. No intra-or extra-axial hemorrhage. No evidence of cytotoxic edema corresponding to a large vascular territory. No acute fracture.

**Figure 2:**
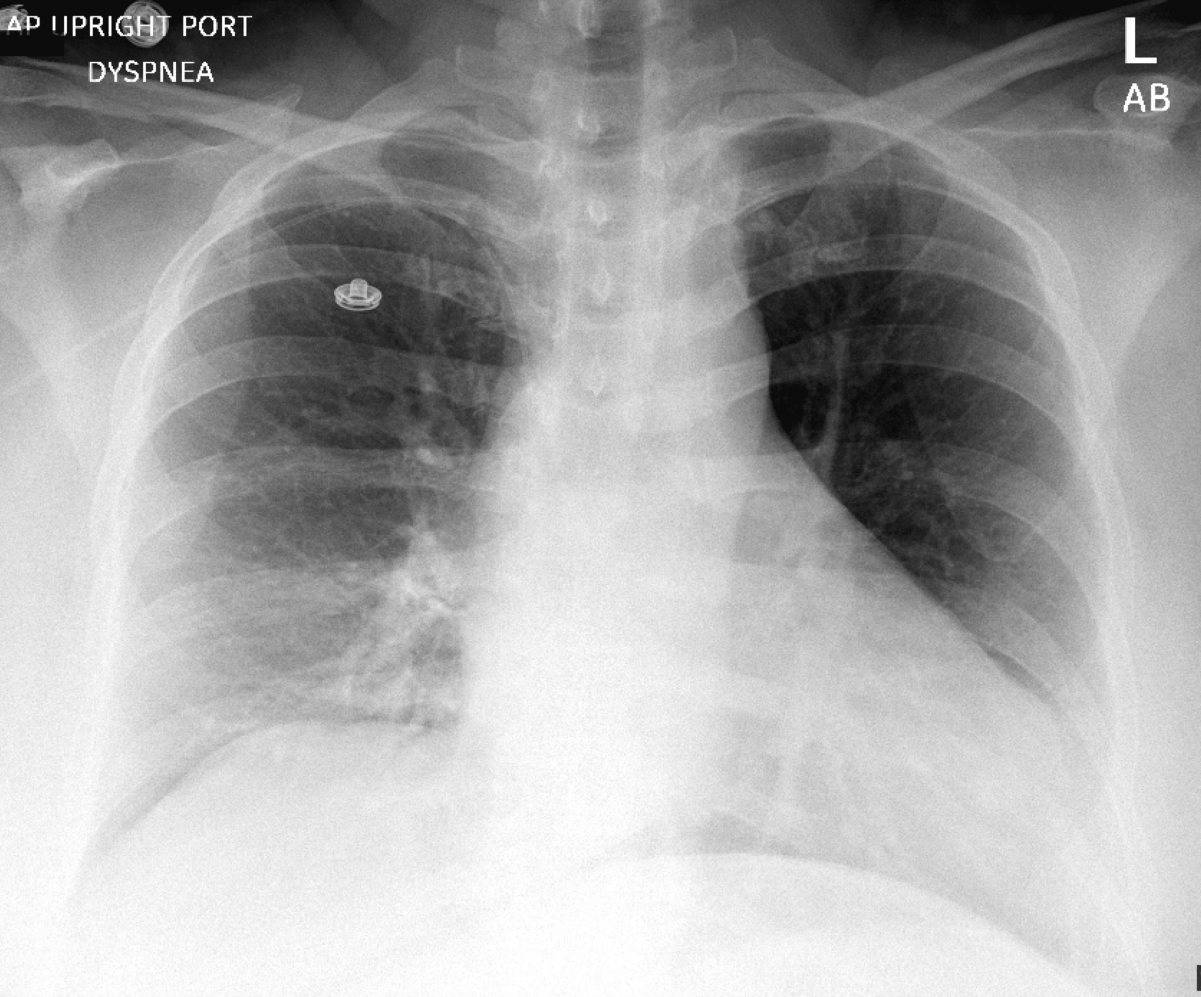
Chest X-ray, anterior posterior view. No pneumothorax or sizable pleural effusions.

On arrival, she was afebrile but met systemic inflammatory response syndrome (SIRS) criteria; she presented with an elevated white blood cell count and tachycardia (Table 1). Serum sodium was decreased (Table 1). Due to her initial presentation, a CT angiography of the head and neck was obtained, which was negative but limited by motion artifact (Figure 1). Chest X-ray was unremarkable (Figure 2). The patient was admitted for further evaluation. Neurology was consulted, who initiated lacosamide but later transitioned her to home divalproex sodium after electroencephalography showed no epileptiform activity. 

**Table 1:** Quantitative laboratory parameters at admission and during the hospital course. Laboratory values are presented with respective reference ranges and qualitative interpretations. Abbreviations: CSF: cerebrospinal fluid, Ag: antigen

Parameter	Value	Reference range	Interpretation	Time
WBC	17.2 × 10⁹/L	4.0–10.5 × 10⁹/L	Increased (leukocytosis)	Admission
Sodium	127 mmol/L	135–145 mmol/L	Decreased (hyponatremia)	Admission
Serum cryptococcal Ag titer	1:1280	Negative	Positive	Admission
CSF WBC	87 cells/µL	≤5 cells/µL	Increased (lymphocytic)	Day 3
CSF protein	28 mg/dL	15–45 mg/dL	Normal	Day 3
CSF glucose	50 mg/dL	40–70 mg/dL	Normal	Day 3
CSF opening pressure	>55 cm H₂O	<20 cm H₂O	Markedly elevated	Day 3
CD4 count	1258 cells/µL	500–1500 cells/µL	Normal	Admission
CD4 %	36%	30–60%	Normal	Admission

Due to the patient's prolonged headache and new-onset seizures, Interventional Radiology (IR) was consulted for an image-guided lumbar puncture (LP). The image-guided LP revealed significantly elevated opening pressure with clear CSF with increased white blood cells, normal protein, and glucose levels within CSF fluid (Table 1). CSF cryptococcal antigen was positive, and culture grew *C. neoformans*; the initial serum cryptococcal antigen was elevated. HIV testing, hepatitis C testing, and quantitative immunoglobulin levels were unremarkable (Table 1). 

**Figure 3:**
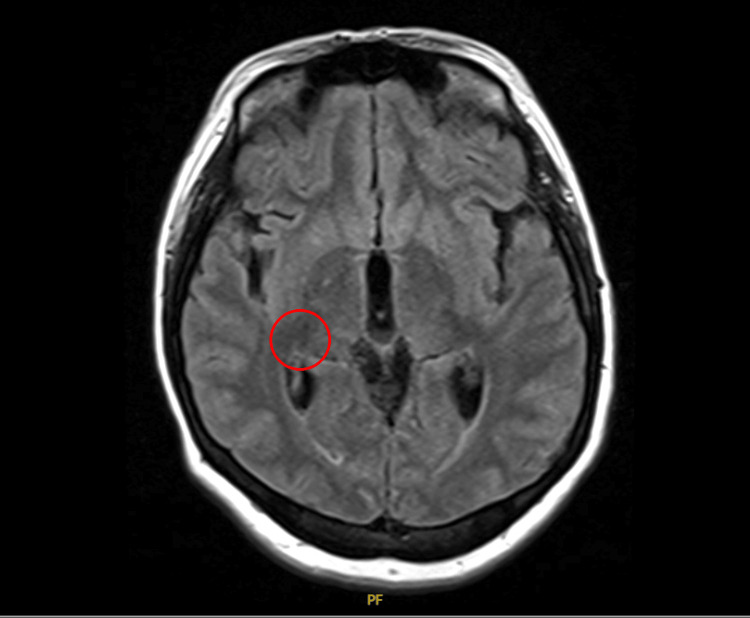
Magnetic resonance image (MRI) of the head, transverse view. Small focus of hypodensity involving the right lentiform nucleus, circled in red, likely from chronic microvascular ischemic disease. No acute infarction. The brain parenchyma appears normal otherwise. No hemorrhage, mass, abnormal fluid collections, midline shift, cerebral infarction, or hydrocephalus.

Liposomal amphotericin B and flucytosine were initiated, and serial therapeutic LPs were performed for ICP control. Ophthalmologic evaluation revealed papilledema and right-eye visual blurring. MRI of the brain demonstrated only small chronic microvascular changes without acute infarction, mass effect, or hydrocephalus (Figure 3). 

**Figure 4:**
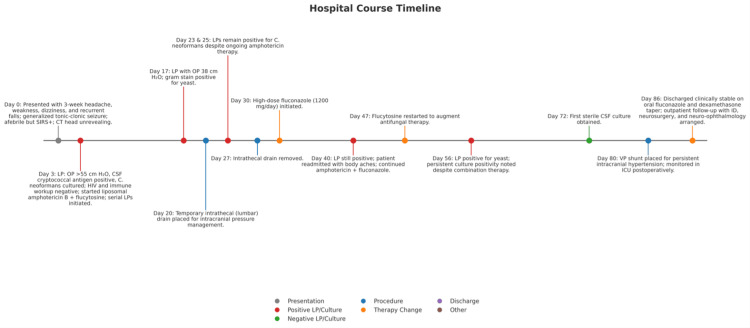
Grpahical timeline showing the patient's hospital course. Gray indicates patient presentation and symptom evolution; red denotes positive CSF studies (LP and/or cultures); green denotes sterile CSF cultures; blue represents ICP-directed procedures; orange corresponds to antifungal therapy initiation or modification; purple denotes discharge disposition; black indicates other documented clinical events.

Despite >6 weeks of amphotericin B (400mg/day) and flucytosine (100 mg/kg/day), serial CSF cultures on hospital days 17, 23, 25, 40, and 56 remained positive for *C. neoformans *(Figure 4). A temporary lumbar drain was placed on hospital day 20 and removed after one week. The first sterile CSF culture was obtained on hospital day 72, approximately two weeks before VP shunt placement on hospital day 80 for persistently elevated ICPs (Figure 4). 

High-dose fluconazole (1200 mg/day) was initiated on hospital day 30 (Figure 4).

Headaches resolved with supportive care; imaging confirmed shunt patency. Renal function remained stable despite prolonged amphotericin B therapy. Ophthalmology follow-up confirmed persistent right-eye blurring and bilateral peripheral microaneurysms. She was transitioned to oral fluconazole (800 mg/day) for consolidation therapy and a dexamethasone taper. She was discharged on hospital day 86 (Figure 4) with outpatient follow-up arranged with infectious disease, neurosurgery, and neuro-ophthalmology. 

## Discussion

This case highlights a rare presentation of CM in an immunocompetent patient. Unlike the typical population of patients with advanced HIV, solid organ transplants, or other immunodeficiencies, our patient was immunocompetent with no evidence of underlying immunodeficiency. 

Current guidelines recommend induction therapy with amphotericin B plus flucytosine for four to six weeks, followed by consolidation with fluconazole [1,7]. Most patients achieve sterile CSF cultures within two to four weeks. By contrast, our patient remained culture-positive for more than 70 days, which is rarely reported in immunocompetent hosts [4,5]. Notably, our patient remained culture positive despite clinical improvement. The persistence of positive CSF cultures for over 70 days despite guideline-directed antifungal therapy gives emphasis to the challenges of managing refractory cryptococcal infections.

The unusually prolonged persistence of positive CSF cultures in this case was likely multifactorial. A high initial fungal burden, medication intolerance requiring adjustment of antifungal therapy, and markedly elevated ICP may all have contributed to delayed CSF clearance.

CM most commonly affects immunocompromised patients; however, disease in immunocompetent individuals has been widely described in the literature. In such cases, presentation may be subacute, and diagnosis is often delayed. Our patient presented with headaches, dizziness, and visual changes, which are consistent with prior reports of cryptococcosis in immunocompetent hosts [4,5]. 

Reviews summarizing multiple cohorts note that 70-80% of CM cases occur in immunocompromised hosts overall, patients with HIV-associated CM typically present in their mid-30s (median 35-38 years), are predominantly male (58-80%), and have profound immunosuppression with median CD4 counts ~20-90 cells/µL, with ~75% presenting below 100 cells/µL [8,9]. On presentation, CM exhibits very high antigen titers, approximately 1:1280, with higher titers being associated with higher fungal burden and mortality [10].

By contrast, our patient was a 40-year-old female who was HIV negative and had a CD4 count of 1258 cells/µL; she was also negative for hepatitis C, further supporting that she was immunocompetent. Autoimmune etiologies were considered but ruled out, as the patient had negative ANA, RF, and ds-DNA testing. DM has been postulated as a risk factor for CM [11]. DM was ruled out in our patient due to a normal hemoglobin A1C and normal blood sugars. 

Elevated ICP management

Elevated ICP is a well-recognized complication of CM, associated with high morbidity and mortality [1,3,12]. Standard management includes serial lumbar punctures, temporary lumbar drains, or VP shunting in refractory cases [6,13-16]. Our patient required multiple lumbar punctures, temporary lumbar drain placement, and ultimately VP shunt placement, which led to the resolution of headaches, but not the resolution of vision. This highlights the importance of timely recognition and escalation of ICP management strategies when the standard fails to achieve the desired therapeutic outcomes. 

Obesity is not a risk factor for CM. However, there have been case reports of obese patients, who are otherwise immunocompetent, developing CM [11]. While there are no studies showing that obesity is a risk factor, our case adds to the growing body of literature suggesting that obesity and metabolic dysregulation play a role in the immune response to CM.

## Conclusions

This case underscores several important lessons. CM can occur in immunocompetent hosts and should remain in the differential diagnosis for patients presenting with subacute meningitis symptoms, even in the absence of traditional risk factors. Persistent positive cultures require individualized management beyond standard guidelines, particularly when antifungal intolerance or complications arise. Aggressive monitoring and timely neurosurgical intervention for ICP management are essential to prevent irreversible neurologic damage. 

In summary, our case highlights the potential for severe, refractory CM in an immunocompetent host, with a prolonged and complex clinical course. These findings emphasize the importance of vigilant monitoring, tailored antifungal therapy, and proactive ICP management to optimize outcomes. Further research is warranted to better define the role of comorbidities such as obesity and diabetes in the pathogenesis and progression of cryptococcal disease.
